# Functional Roles of FGF Signaling in Early Development of Vertebrate Embryos

**DOI:** 10.3390/cells10082148

**Published:** 2021-08-20

**Authors:** Vijay Kumar, Ravi Shankar Goutam, Soochul Park, Unjoo Lee, Jaebong Kim

**Affiliations:** 1 Department of Biochemistry, Institute of Cell Differentiation and Aging, College of Medicine, Hallym University, Chuncheon 24252, Korea; vijay10187@gmail.com (V.K.); ravi2005gautam@gmail.com (R.S.G.); 2 Department of Biological Sciences, Sookmyung Women’s University, Seoul 04310, Korea; scpark@sookmyung.ac.kr; 3 Department of Electrical Engineering, Hallym University, Chuncheon 24252, Korea; ejlee@hallym.ac.kr

**Keywords:** FGF, FGFR, embryonic development, germ layer formation, transcription regulation, embryonic patterning

## Abstract

Fibroblast growth factors (FGFs) comprise a large family of growth factors, regulating diverse biological processes including cell proliferation, migration, and differentiation. Each FGF binds to a set of FGF receptors to initiate certain intracellular signaling molecules. Accumulated evidence suggests that in early development and adult state of vertebrates, FGFs also play exclusive and context dependent roles. Although FGFs have been the focus of research for therapeutic approaches in cancer, cardiovascular disease, and metabolic syndrome, in this review, we mainly focused on their role in germ layer specification and axis patterning during early vertebrate embryogenesis. We discussed the functional roles of FGFs and their interacting partners as part of the gene regulatory network for germ layer specification, dorsal–ventral (DV), and anterior-posterior (AP) patterning. Finally, we briefly reviewed the regulatory molecules and pharmacological agents discovered that may allow modulation of FGF signaling in research.

## 1. Introduction

**Figure 1:**
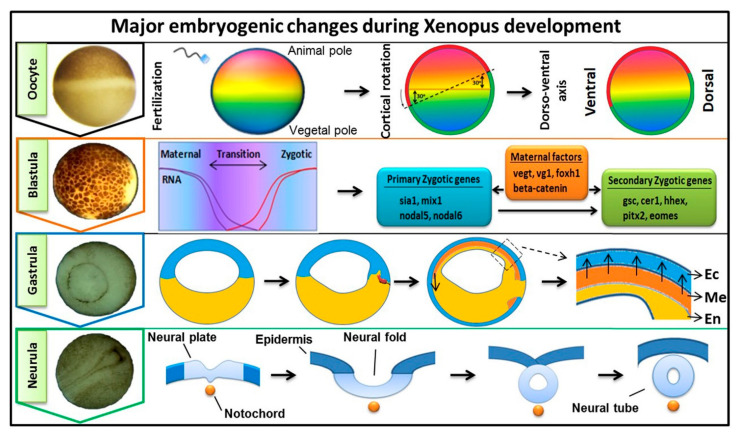
Key developmental stages and major events during oocyte, blastula, gastrula, and neurula. Oocyte: sperm entry establishes the DV (dorsal-ventral) axis. Blastula: mid-blastula transition (MBT) whereby expression of zygotic genes begin with maternal factors inducing primary zygotic and secondary zygotic genes. Gastrula: a cluster of dorsal mesodermal cells start their migration and form the three primary germ layers (Ec (ectoderm), Me (mesoderm, arrow head indicates the secretion of extracellular proteins such as Chordin), and En (endoderm)). Neurula: formation of the neural tube and AP axis establishment.

Early embryogenesis in vertebrate embryos involves the irreversible developmental process. As the ovum receives the male haploid genome from a sperm to become a diploid cell, the process of fertilization is started with the fertilized ovum then being referred to as a zygote. The zygote goes through several key developmental stages including mid-blastula transition (MBT), gastrulation (germ layer formation), and neurula, for establishing the overall body axis and generating the anterior CNS and posterior PNS (Figure 1). These are tightly controlled spatiotemporal events led by several signaling pathways and occur in conjunction with maternal or zygotic morphogen gradients throughout the embryos. FGF signaling is known to play an essential role during embryonic development [1,2,3], and in this review, our discussion was focused on involvement of FGF signaling in early embryogenesis. The first discovered FGF ligand, FGF2 (also known as basic FGF/bFGF), was purified from brain tissue in 1975 and was defined for its stimulatory activity in fibroblasts [4]. Since then, a total of 22 FGF members have been identified in humans and similar numbers in vertebrates. Except for the intracellular FGF11 subfamily, these interact with particular FGF receptors (FGFRs) to activate intracellular effector proteins. FGF/FGFR signaling regulates a plethora of cellular processes mediated by activation/modification of cytosolic effectors and followed by transcriptional regulation of target genes. Dysregulation of FGF signaling has been reported to promote several human diseases and disorders, and their severity can vary based on active ligands/receptors and tissues involved [5]. Several lines of evidence also indicate a crucial role for FGF signaling in early embryogenesis for germ layer formation [1,2] and organogenesis [6]. During primordial germ layer specification, FGFs modulate fate determination as autocrine and paracrine signaling agents. Alterations in tightly regulated FGF expression patterns, as with altered FGFR splicing or mutation and changes in spatiotemporal FGF-FGFR interactions, may result in flawed and defective development for multiple congenital diseases and the onset of various cancer types [5,7,8]. In FGF/FGFR genes, genetic mutations that lead to several congenital diseases have been described reviewed elsewhere [9]. In this review, we summarized the information and our understanding of the functional role(s) of FGFs in early embryonic germ layer specification and axis formation during embryogenesis.

## 2. FGF and FGFR Families and Signal Transduction

### 2.1. FGF Ligands

The FGFs are a large family of growth factors consisting of 22 members in humans and mice, and 19 members identified in Xenopus (Table 1). In this family, there are seven subfamilies described in vertebrates, namely FGF1, FGF4, FGF7, FGF8, FGF9, FGF11, and FGF19 (reviewed [10,11]). Subfamily members have high similarity in their amino acids sequences. Except for the intracellular FGF11 subfamily, extracellular secretion of a given FGF is required for its signaling and function. Based on their secretion profile, FGF members can also be placed into two groups. The first are those whose secretion takes place through a classic endoplasmic reticulum-Golgi secretion pathway in the cells as these FGFs contain *N*-terminal hydrophobic peptides and they include FGF3, 4, 5, 6, 7, 8, 10, 17, 18, 19, 21, and 23 [10]. The second group of FGFs are those that do not contain *N*-terminal hydrophobic peptides and are endoplasmic reticulum-Golgi-independent for their secretion. These include FGF1, 2, 9, 16, and 20 [10,11,12]. As an exception, FGF22 remains attached to the cell surface by its *N*-terminal signal peptide rather than being secreted (reviewed [10]). All members of FGF11 subfamily (Table 1) are known as non-secretory FGFs and are strictly intracellular proteins. Even though these FGFs share structural homologies with other secreted FGFs, they do not share any functional similarities [13,14]. As intracellular entities, FGF11 subfamily members have been documented as being components of certain protein kinase-mediated signaling pathways and they also interact with membrane channels to regulate cell fate [15,16].

### 2.2. FGF Receptors

FGFs interact with specific FGF receptors to initiate intracellular signaling and there are four cell membrane tyrosine kinase FGF receptors, FGFR1, 2, 3 and 4, which are members of the larger receptor tyrosine kinase (RTK) group [17,18]. Each FGFR is a single-pass transmembrane (TM) protein that includes an *N*-terminal extracellular ligand FGF binding domain and a *C*-terminal intracellular tyrosine kinase domain. The extracellular domain contains 3 immunoglobulin-like subdomains (D1, D2, and D3 domains) [19]. There is an also an acidic box between D1 and D2 domains. The D2 and D3 domains facilitate FGF binding [19]. Heparan sulfate (HS) is a coreceptor for FGF binding to an FGFR, and it is essential for FGF binding and signaling. HS is one of the abundant polysaccharides found in the extracellular matrix of mammalian cells [20], and it interacts with the cationic patch found in both FGF and D2 subdomain of FGFR [20]. Ligand binding induces a conformational change in FGFR, leading to its dimerization and activation of the its intracellular kinase [21]. For FGFR1, FGFR2, and FGFR3, two standard isoforms (b-and c-isoforms) are generated by splicing [22]. The splicing variants have altered ligand affinity for various FGFs, except for FGF1, acknowledged as a universal ligand and being able to interact with both FGFR isoforms [17,20].

### 2.3. An Overview of FGFR Signaling Pathways

In this section, we briefly summarized the activation modes of FGFR associated cytosolic effectors and linked components that act as intermediates. Several partner receptor proteins may be associated with the cytosolic domain of an FGFR, such as cell adhesion molecules (CAMs) and G-protein coupled receptors (GPRCs) [23]. Signal-induced activation of FGFR typically activates multiple cytosolic signaling pathways. An FGFR is mainly associated with its intracellular signaling intermediates, including phospholipase C (PLCγ), FRS1, FRS2/FRS2α, and FRS3/FRS2β (reviewed [24]). FRS2 recruits the adaptor GRB2 (growth factor receptor-bound 2) [25], and once GRB2 is bound to the functional domain of the FRS2, it can interact with either SOS or GAB1 and form a complex [26]. Upon FGF ligand binding to FGFR and heparan sulfate, multiple cytosolic events occur; these are mostly activational phosphorylations. Once the FGFR complex is activated, GRB2/SOS exchanges the GDP to GTP for Ras; GTP-Ras in turn activates and stimulates Raf (also known as MAPK kinase kinase) as part of the Ras/MAPK pathway (for a detailed mechanism, refer to reviews [27,28,29,30]). Similarly, GRB2 switches on PI3K/Akt signaling cascade, as activation of PLCγ/PKC and JAK/STAT pathways are also directly linked to FGFR activation [27,28,29,30]. These pathways generate the signals leading to targeted transcription factors regulating transcription of their target genes.

**Table 1:** Known FGFs and FGFRs in human, mice, and Xenopus.

	Human		Mouse		Xenopus	
Subfamily	Members (Other Name)	Ref.	Members (Other Name)	Ref.	Members (Other Name)	Ref.
FGF1	FGF1 (aFGF)	[31]	FGF1 (FGFa)	[32]	xFGF1	[33]
	FGF2 (bFGF/FGF-β)	[4]	FGF2	[34]	xFGF2 (bFGF)	[35]
FGF4	FGF4 (eFGF)	[36]	FGF4 (KFGF)	[37]	xFGF4 (eFGF, fgf4-a, fgf4-b)	[38]
	FGF5	[39]	FGF5	[40]	xFGF5	[33,41]
	FGF6 (HST2, HSTF2)	[42]	FGF6 (HSTF2)	[43]	xFGF6	[33,41]
FGF7	FGF3	[44]	FGF3 (Int-2)	[45]	xFGF3 (INT-2, FGF3A)	[46]
	FGF7 (KGF)	[47]	FGF7 (KGF)	[48]	*	
	FGF10	[49]	FGF10	[50]	xFGF10	[51]
	FGF22 (UNQ2500/PRO5800)	[52]	FGF22	[52]	xFGF22	[41]
FGF8	FGF8 (AIGF)	[53]	FGF8 (AIGF)	[54]	xFGF8 (FGF8a, FGF8b)	[55]
	FGF17 (UNQ161/PRO187)	[56]	FGF17	[56]	*	
	FGF18 (UNQ420/PRO856)	[57]	FGF18	[57]	*	
FGF9	FGF9	[58]	FGF9	[59]	xFGF9 (GAF, HBGF9)	[60]
	FGF16	[61]	FGF16	[62]	xFGF16	[41]
	FGF20	[63]	FGF20	[64]	xFGF20	[65]
FGF11	FGF11 (FHF3)	[66]	FGF11 (FHF3)	[66]	xFGF11	[41]
	FGF12 (FGF12B, FHF1)	[66]	FGF12 (FHF1)	[67]	xFGF12	[68]
	FGF13 (FHF2)	[69]	FGF13 (FHF2)	[67]	xFGF13	[70]
	FGF14 (FHF4)	[71]	FGF14 (FHF4)	[72]	xFGF14 (FHF4)	[33]
FGF19	*		FGF15	[73]	*	
	FGF19 (UNQ334/PRO533)	[74]	*		xFGF19	[41]
	FGF21 (UNQ3115/PRO10196)	[75]	FGF21	[75]	*	
	FGF23 (HYPF, UNQ3027/PRO9828)	[76]	FGF23	[76]	xFGF23 (fgf23.1, FGF23.2)	[41]
	FGF receptor family					
	FGFR1	[31]	FGFR1	[77]	xFGFR1	[78]
	FGFR2	[79]	FGFR2	[80]	xFGFR2	[81]
	FGFR3	[82]	FGFR3	[83]	xFGFR3	[84]
	FGFR4	[85]	FGFR4	[86]	xFGFR4	[87]

Asterisk (*) represent unidentified.

## 3. FGF Signaling in Embryonic Germ Layer Formation

**Figure 2:**
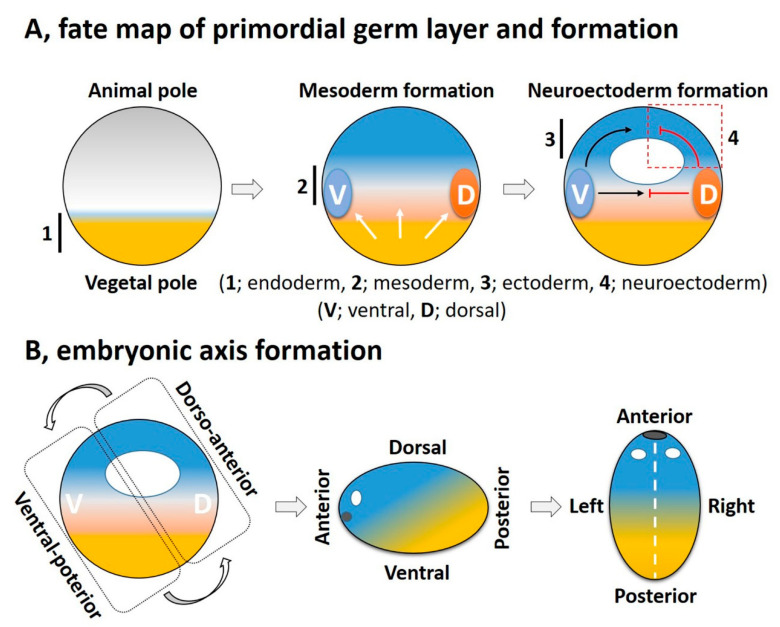
The fate map and key steps in overall embryonic germ layer and axes formation. (**A**) Vegetal pole (endoderm) contains higher amounts of maternal factors that generate the mesoderm inducing signals (white arrows). These mesoderm inducing signals establish the ventral (V) and dorsal (D) signaling centers. Finally, dorsal signals (BMP antagonists) antagonize the ventral and allow the neuroectoderm formation (red dotted box), as ventral signaling commences the ectoderm (epidermis) specification. (**B**) Sequential steps in embryonic axes formation: Dorsal and ventral halves extend to give the anterior and posterior axes, respectively. The left–right (LR) axis establishes the neurula stage embryos by a symmetry breaking signal. The LR axis is generally described by positioning of the visceral organs along the midline (white dotted line).

Cellular diversification is an essential process in generating a complex, multicellular organism. This process begins with gastrulation as the three germ layers of endoderm, mesoderm, and ectoderm are specified. For an amphibian embryo, the first embryonic germ to specify is the endoderm from the vegetal pole. The formation of mesoderm, the second germ layer, is the result of active mesoderm inducing signals, originating from the vegetal region or the prospective endoderm, which triggers the mesoderm specification. During the mesoderm specification, generally two distinct signaling centers, namely the ventral and dorsal signaling centers, are established in the marginal region; these are based on presence and activation of maternal or zygotic factors at ventral and dorsal half of the embryo (Figure 2A). Once the ventral and dorsal signaling centers are established, they both collectively drive further germ layer specification. The dorsal centers (also called the dorsal mesoderm, dorsal organizer, and organizer) produce dorsal mesoderm and promote neuroectoderm formation by inhibiting ventral signaling. On the opposite end, the ventral center provides the ventral mesoderm and the ectoderm (for epidermis formation) (Figure 2A). In this section, we try to summarize primordial germ layer formation and the active role of FGF signaling in this process.

### 3.1. Role of FGF Signaling in Endoderm Formation

**Figure 3:**
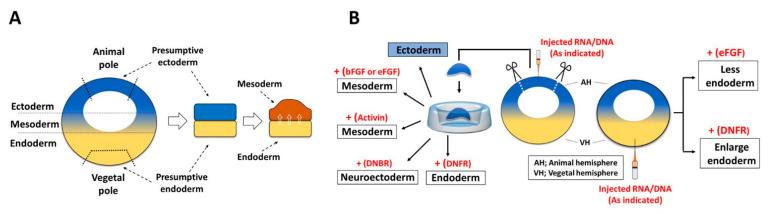
Outline of key experiments in embryonic induction. (**A**) a conjugation assay for vegetal explant and ectodermal explant. (**B**) The embryos are injected with RNA/DNA of various genes (indicated in red text) and the dissected explants are then cultured. Upon injection of an inducer, the explants then differentiate into various embryonic germ layers (indicated in boxed text). The majority of experiments are performed with Xenopus embryos.

In amphibian embryo, the vegetal half (vegetal pole) contains several important maternal factors (also called as vegetal factors) that might govern early primordial germ layer formation. To date, several transcription factors primarily located in the vegetal region have demonstrated consistent inductive activity for germ layer differentiation. One instance is Vegt (also known as Xombi and Brat), which is a maternal T-box transcription factor as depleted *vegt* abolished endoderm formation and overall germ layer patterning [88]. Similarly, blocking of Vg1, one of the maternal (TGFβ family) vegetal factors, severely curtailed endoderm and mesoderm (mostly for dorsal organizer) development in Xenopus embryos [89]. These results indicate that Vegt and Vg1 are important, necessary factors for endoderm formation. Depleted *vegt* dramatically reduced the expression of *fgf3*, *fgf4*, *fgf8*, *Xnr1*, *Xnr2*, and *Xnr4*; however, expression of *bmp4* and *bmp7* remained unaffected or were increased [90]. FGF signaling then showed an inhibitory effect on endodermal differentiation and blocking FGF signaling with DNFR in ectodermal explants induced endodermal specific genes *endodermin* (*edd*) and *mixer* expression [91]. Recently, Dusp1 (dual specificity phosphatase 1, a FGF signaling modulator) reported to increase the expression of *edd*, *mixer*, and *sox17β* (endodermal marker) in a activin/smad2-dependent manner [92]. The one possible explanation of this observation may be additional expression of dusp1 with smad2 might inhibit the FGF signaling and indirectly induces smad2 mediated endodermal differentiation. Similarly, morpholino based knockdown of Dusp4 (a MAP kinase inhibitor) abolishes the expression of *sox17* and endodermal defects [93]. Similarly, DNFR injection in the vegetal region led to enlarged endodermal tissue in whole Xenopus embryos (Figure 3B) [91]. There have been supporting reports on zebrafish embryos, where FGF/ERK signaling leads to inhibitory phosphorylation of Sox32 (an endodermal specifier), and thus not being able to induce *Sox17* [94]. These set of experiments implied that FGF signaling may be required for limiting endodermal genes expression in a negative feedback loop, and that FGF levels may need to be below a certain threshold for endoderm to proceed.

The role of FGF signaling, however, is not always endoderm inhibitory, at least in certain systems. From stem cell research, FGFs cooperate with activin signaling in promoting endodermal differentiation as the down-regulation of FGF signaling by SU5402 (an FGF inhibitor) abolished activin-A induced definitive endoderm formation in human embryonic stem cells (hESCs) [95]. The activin-A treatment induces definitive endoderm expression additively when FGF2 is additionally supplied to the culture media. In this system, activin-mediated definitive endoderm induction required FGF signaling, but the converse was also true as FGF2 failed to induce definitive endoderm under activin depleted conditions [95]. These effects indicate that definitive endoderm differentiation might be a combined effect of activin and FGF signaling in hESCs. Several other maternal or zygotic transcription factors have also been reported to induce endodermal fate, for example Otx1, Sox7, and Sox17 (reviewed in [96]). These research findings point to endoderm fate specification being the result of coordinated signaling from distinct pathways, but the role and conditions of FGF signaling in whether being antagonistic or additive/synergetic in endoderm formation is poorly understood and requires further analysis.

### 3.2. FGF in Mesoderm Induction

In 1969, an inductive role for endoderm in inducing mesoderm formation was demonstrated. In this experiment, the section from the animal half (animal explant or animal caps) and the section from vegetal half were conjugated and cultured [97]. Surprisingly, an animal cap keeps ectodermal identity autonomously; in the conjugation condition, it converts to a mesodermal tissue, while a vegetal explant keeps its original identity and remains endoderm (Figure 3) [97]. Later on, bFGF (FGF2) or eFGF (FGF4) were implicated in inducing mesoderm in animal cap explants of Xenopus embryos, mimicking vegetal explant activity [91,98]. A similar finding was demonstrated in rabbits [99] and mice [100], where FGF2 sufficiently induced mesoderm differentiation. In 1991, when Amaya et al. injected a dominant-negative mutant of the FGF receptor (DNFR/XFD) into Xenopus embryos, ectopic expression of DNFR sufficiently blocked the wild-type FGFR mediated signaling [101]. The DNFR injected embryos completely failed to produce mesodermal tissue [101]. In mice, targeted point mutation of FGFR1 led to embryos exhibiting several subtypes of mesodermal defects [102]. Since then several independent studies have provided crucial evidence indicating FGF signaling as an important instructive factor in mesoderm differentiation of vertebrates [101,102,103,104,105].

The active molecules with mesoderm inductive activity from the vegetal hemisphere include members of Xenopus nodal-related factors (Xnr1, 2, 4, 5, and 6) and TGFβ family (activin βB), now known as endogenous mesoderm inducing factors in various animal models, including Xenopus, zebrafish, and mouse [106,107,108,109] and the signaling pathways utilized by these signals are often due to activin ligands [106,107,108,109]. This mesoderm inducing effect by activin treatment in metazoan embryogenesis is now widely accepted. The ectopic expression of activin/Nodal downstream intracellular effectors Smad2/3 activate *xbra*, *chordin (chrd)*, *noggin (nog)*, and *goosecoid (gsc)* expression [110]. Activin-mediated mesoderm induction critically depends on FGF signaling, as demonstrated when DNFR led to complete loss of mesodermal genes expression due to activin [111]. In comparison, ectopic expression of Smad2 significantly elevates expression of various FGFs and FGFRs including *fgf3*, *fgf8*, *fgf20*, *fgfr1*, and *fgfr2* [92]. Supporting the role of FGFR signaling in mesoderm induction is also observed with the expression profile of several FGF ligands in early mesoderm of vertebrate embryos. *Fgf3* expression is primarily in the ring around blastopore lip (mesoderm) of Xenopus gastrula, which then resolves into neural or brain tissues at a later stage of development [112]. Similarly, *fgf4*, *fgf8*, and *fgf20* are largely expressed in early mesoderm or late mesodermal lineages [113,114,115,116]. However, integration of FGF and activin/nodal signaling in mesoderm specification is a potential subject for a future investigation.

Brachyury (Bra/Xbra) is a T-box transcription factor and signature mesodermal (also known as a pan-mesodermal marker) factor, and is actively involved in gene regulation process required for mesoderm induction and differentiation [117,118]. Similarly, Eomesodermin (Eomes) is another T-box transcription factor, an important mesodermal factor crucial for mesoderm induction and differentiation [119]. Both proteins are necessary for mesoderm induction; however, Xbra is also involved in expression of ventral/posterior specific genes (e.g., *ventx1.1* and *xhox3*) [117,120,121,122]. Xbra is indispensable for mesoderm formation and maintenance since knockdown of Xbra converts its mesodermalizing character to a neuralizing one [123,124]. Eomes has been reported to induce dorsal/anterior mesodermal (*gsc*, *chrd*, and *nog*) genes in Xenopus embryos [119]. Xbra early expression was reported to depend on FGF signaling as embryos treated with SU5402, showed no *xbra* expression [114]. In turn, FGF2 and FGF4 have been reported to induce *xbra* transcription in animal explants of Xenopus embryos, wherein Xbra makes an autocatalytic regulatory loop to maintain the *Fgf4* expression [123,125]. Xbra can maintain the expression *fgf4*, but its activation is most likely thought to depend on Nodal signaling; this is similar to *fgf8* activation largely depending on nodal/activin signaling [114]. FGF8, have two alternative splice variants (protein isoforms), namely FGF8a and FGF8b, and these show different inductive features. FGF8a has been reported to have mostly neural inducing activity with little mesoderm inducing capability, whereas FGF8b is important for mesoderm induction and differentiation [55].

FGF signaling has an indispensable role in mesoderm induction and specification, but the detailed molecular mechanisms by which different FGF ligands regulate a different subset of mesodermal markers is not fully understood. Based on their spatiotemporal expression patterns, these markers may be divided as dorsal, ventral, and pan-mesodermal markers. In dorsal mesoderm of Xenopus gastrula, *chrd*, *nog*, *gsc*, and *siamois (sia)* are highly expressed in dorsal mesoderm [126], in which *gsc* expression is sustained under FGF inhibitory conditions [127]. However, *chrd* and *nog* (from the organizer) have been reported to be both sensitive and insensitive to FGF signaling, thus complicating the interpretation of their underlying mechanism [127,128]. As Nodal/activin signaling also plays an upstream role for various mesodermal genes [103,107,109,110], the exclusive involvement of FGF signaling at a transcriptional level for these set of genes is not fully understood.

With the early ventral mesoderm, which later leads to development of posterior tissues, several pieces of evidence indicate that FGF signaling plays an upstream role to the RAR (retinoic acid receptor) [129]. In particular, RARα2 has been reported to cooperate with FGF signaling and also required for normal expression of FGF8, FGFR1, and FGFR4 [129]. RARα2 and FGFs are collectively required for *xcad3* and *hoxb9* (posteriorizing factors) expression and normal axis development in Xenopus [129]. Cdx (caudal type homeobox) transcription family are also known to be transcriptionally activated by FGF signaling and be necessary for proper dorsoventral axis formation [130,131,132]. A further crosstalk of various pathways involves FGF dependent Xbra physically interacting with Smad1 to activate the expression of *ventx1.1*, a ventral mesodermal/ectodermal inducer and a BMP/Smad1 target transcription factor [120].

A spatiotemporal expression of FGF ligands and receptors is seen in Xenopus embryos, suggesting diverse expression patterns among ligands. During gastrulation, *fgf1*, *fgf2*, *fgf4*, *fgf8*, *fgf20*, and *fgf22* are highly expressed, implying a requirement for mesoderm specification and extension [33]. Several transcription factors have been reported to incorporate FGF signaling in mesoderm specification and embryonic patterning. As an example, mef2d is transcriptionally activated by FGF4 and FGF8 in the marginal region of Xenopus embryos, and from the vegetal region, nodal (Xnr5 and 6) also induces *mef2d* expression. Once Mef2d protein is made, it can form a positive expression feedback loop with FGF4 and FGF8, leading to the expression of mesodermal genes like *xbra*, *chrd*, *gsc*, and *nog* [133]. A similar example of interaction with FGF signaling is for Egr1, which has been reported as a downstream target of FGF signaling and plays an important role in embryonic development. The Egr1 activates the transcription of *myod*, and represses *xbra* transcription [134]. Collectively, the presented evidence suggests that FGF signaling may interact with several other signaling pathways (e.g., activin, nodal, BMP, and RAR) during mesodermal differentiation in a context-dependent manner (for example in the ventral and dorsal mesoderm). However, the details for the different ligands, their receptors, and their mechanistic integration with other key signaling entities remain to be fully elucidated.

### 3.3. FGF in Ectoderm Specification

BMP signaling is essential for ectoderm (epidermal) specification during embryonic development as BMPs activate ectoderm specifier *ventx1.1*, *ventx1.2*, and *ventx2.1* via the Smad1 pathway to derive epidermal fate [135,136]. Previous studies indicate that FGF signaling antagonistically interacts with BMP signaling and inhibits ectoderm differentiation, particularly in the dorsal region of the embryo. The evidence suggests that FGF signaling induces linker region phosphorylation of Smad1 with an inhibitory phosphorylation and reduces *C*-terminal activational phosphorylation [120,137,138]. In Xenopus, blocking BMP signaling also induces *fgf4* expression [139]. In zebrafish blastula, FGF restricts the *bmp2* and *bmp7* expression in the ventral region, promoting the dorsal fate in the BMP inhibited condition [140]. Also in zebrafish, inhibition of FGF signaling extends the BMP activity and generates ventralized embryos [140], again supporting the inhibitory role of FGF in ectoderm formation. FGF signal is inhibitory to BMP/Smad1 in ectoderm specification. However, FGF/Xbra functions synergistically to BMP/Smad1 in ventral mesoderm specification. Interestingly, recent studies suggest that FGF signaling support ventral mesoderm formation. In Xenopus gastrula, FGF downstream target, *xbra*, a pan mesodermal marker, cooperates with Smad1 to activate *ventx1.1* transcription [120], and Xcad2, an FGF downstream target, induces the *ventx1.1*, *ventx1.2*, and *ventx2.1* expression [130]. These studies demonstrate that the function of FGF in ectoderm and ventral mesoderm specification occurs in two ways: first, as being directly inhibitory to BMP/Smad1 pathway via phosphorylation modification of Smad1, and second, being ventral mesoderm promoting with increasing of *ventx1.1*, *ventx1.2*, and *ventx2.1* (ventral mesoderm markers) levels via Xbra and Xcad2. These findings imply a context-dependent (dorsal vs. ventral region of the embryo) function for FGF on ectoderm formation. The regulation of each modality, however, remains to be further studied.

### 3.4. Role of FGF in Neural Induction and Neuroectoderm Formation

Neuroectoderm formation starts from the superficial ectodermal layer near the organizer [141], in a region known as Henson’s node in avian embryos [142], and the embryonic shield in zebrafish [143]. From the organizer, neural inducer activity was first demonstrated for Nog [144] and Chrd [145]. These molecules were first believed to provide direct inductive signals; however, later studies confirmed them as BMP inhibitors in a default model of neurogenesis (reviewed [146,147]), where inhibition of BMP signaling is sufficient to induce neurogenesis. Of the many experiments that supporting this model include the knockdown and deletion of *chrd*, *nog*, and *Follistatin*, abolishing the neural plate formation [148]. Triple knockdown or deletion of BMP antagonists allow BMP and target genes to be robustly expressed, resulting in a complete neural loss. In the converse experiment, however, BMP inhibition was not sufficient for neural fate acquisition. It is concluded that although BMP inhibition is necessary, several inductive signals direct the neural fate in parallel with BMP inhibition [149,150]. This is demonstrated with overexpression of DNFR, dominant-negative FGF receptor, abolishing the neural inducing ability of Chrd and Nog [151,152].

Requirement of FGF signaling in neuroectoderm formation could be considered in two different ways: first, being an instructive signaling, in addition to BMP inhibition, FGF signal itself induces neuroectoderm via activation of neural specific genes. Second, being inhibitory to BMP signaling, to guarantee the neuroectoderm formation, FGF and Map kinase participate in inhibition of Smad1 via linker region phosphorylation. Both ways may function together in neuroectoderm formation. However, the first one is essential and the second one is an additional one since the BMP inhibited condition still requires an FGF input. This implies that intact FGF signaling is necessary as an instructive signal in addition to BMP inhibition for neural fate. Although which FGF member(s) instructs naive ectoderm to neuroectoderm remains to be investigated further, ectopic expression of FGF2 induces the neural genes *zic3* expression in animal explant of Xenopus embryos [123]. Another evidence for a role of FGF signaling in this context was with FGF4 signaling shown to activate early neural marker genes *zic3* and *foxd5a*; with BMP inhibition. Z*ic1* transcription was also increased [139]. BMP signaling inhibited condition also led to increased levels of FGF4, reinforcing the role of FGF signaling in neural induction [139]. This observation raises the possibility of FGF4 involvement in early neural induction and not for the later stages for neural lineages.

More recently, Foxd4l1.1, an immediate early neural marker, was shown to be induced following inhibition of BMP signaling [153]. Foxd4l1.1 in naive neuroectoderm can induce expression of *fgf8a/b* in animal explants without *xbra* and *fgf4* expression [153]. FGF8b contains Smad1 inactivation activity via linker region phosphorylation. FGF8 has been reported to induce the expression of *Xsox3* and *N*-tubulin via FGFR4a signaling [154]. In mice, FGF signaling is also critically required for neural stem cell maintenance and neurogenesis [155]. Similar findings have also been reported in chick embryos [156] again implying a role for FGF signaling in neuroectoderm formation. However, FGF8 may be necessary for further neural development in vertebrates. Indeed, a number of studies have shown a plausible role for FGF8 in later stages, such as midbrain development in chick embryo [157], in retina formation also in chick model [158], and anteroposterior pattering in Xenopus [55]. Together, it still remains largely unknown which FGF member has a role as an endogenous instructive signal to induce neuroectoderm without BMP signal inhibition.

Xenopus embryos provide an easy and powerful tool to investigate the regulatory mechanisms in germ layer specification. In this system, the ventral region (A4 blastomere) of 16 or 32 cell-stage embryo fate mapping has this region differentiate into epidermal cells [127,149]. However, A4 blastomere cells are capable of neural fate acquisition but they do not achieve neural fate in BMP inhibited conditions [127,149]. These results again demonstrate that BMP inhibition is not sufficient to induce neural fate. FGF signal manipulation in this region was then performed by injection of a low amount of FGF4 along with dominant-negative BMP receptor (DNBR); this was able to induce direct differentiation to neural tissue instead of epidermis [127,149]. This study raises a question whether FGF4 is enough to induce neuroectoderm from naïve ectoderm. The basic and embryonic FGF (FGF2 and FGF4) would be sufficient for neuroectoderm formation since FGF/map kinase inhibits BMP/Smad1 signal via phosphorylation of linker region. However, various studies indicate that FGF2/4 alone does not induce neurogenesis in ectodermal explants of Xenopus embryos [38,123,159,160]. A direct target transcription factor gene (ventx1.1, PV.1) of BMP/smad1 functions as neural repressor [136,137] whose transcription is also synergistically increased by FGF target gene *xbra* [120]. Knockdown of ventx1.1 induces neuroectoderm in FGF2 treated ectoderm [123]. The requirement of an FGF signal in addition to the inhibition of BMP for neuroectoderm specification remains to be clarified to resolve the long controversy between the neural induction and default neurogenesis models. In addition, FGF2, FGF4, FGF8, and Foxd4l1.1 may have different neural target genes but they share remarkable similarity in inhibiting BMP signaling for R-Smad(s) by generating inhibitory phosphorylation of the Smad linker region [55,123,139,149,153,158,160]. Collectively, FGF signaling is important for modulating BMP signaling in neural induction; however, additional details for various FGF ligands and their FGFRs and their individual mode of action are needed for a more complete picture to emerge.

## 4. FGF Signaling Integrates with Other Signaling Pathways in a Crosstalk for Embryonic Axis Formation

For an amphibian embryo, the first embryonic axis is the dorsoventral (DV) axis that is established prior to germ layer formation. The anteroposterior (AP) axis is an extension of the DV axis, in which the dorsal expands to the anterior, and the ventral expands to give the posterior end (Figure 2B). The left-right (LR) axis is last to establish in the amphibian embryo (Figure 2B). The FGF signaling or its crosstalk with other signaling pathways orchestrate the overall embryonic patterning. In this section, we discuss the interplay of FGF with other signaling pathways for axis determination.

### 4.1. The DV Axis

At late blastula and early gastrulation, Xenopus embryos exhibit a dorsoventral (DV) axis that is established prior to mid-blastula transition (MBT) and it involves shifting maternal morphogens or molecular gradients in a process known as cortical rotation (Figure 1). The ventral side is sensitive to BMP signaling and inhibition of BMP signaling expands the dorsal axis. Experiments with downregulation of BMP signaling were performed by application of DNBR [161] or using Smad5-somitabum (an anti-morphic Smad, or dominant negative R-Smad) [139]. These induced dorsal axis or dorsal targeted gene expression. Similarly, an anti-morphic PV.1 (Ventx1.1) triggered higher expression of dorsal marker *gsc*, *chrd*, *follistatin*, and *xnot*, resulting in formation of the secondary axis [162]. We have previously discussed (Section 3.2, “FGF in mesoderm induction”) downstream targets of FGF signaling such as *xbra* and *xcad2* known to promote ventral/ectodermal fate via activation of *ventx1.1* expression [120,130]. On the other hand, there is evidence supporting FGF signaling in actively promoting the dorsal mesodermal fate. FGF4, FGF8, and FGF20 are highly expressed in the dorsal animal and marginal region of mesoderm while being absent in the ventral vegetal areas [33,38,115]. There has also been documentation of ERK (FGF dependent) activation in the dorsal animal region of gastrulation stages [163,164,165]. There are also indications that FGF-mediated signaling is not a sole regulator of the DV axis as it mutually cooperates with several other signaling modalities such as BMP, activin, nodal, and Wnt/β-catenin (reviewed in [166]).

### 4.2. The AP Axis

The dorsoventral (DV) axis typically expands to continue with anteroposterior (AP) axis development. The dorsal animal region reaches to the anterior and the ventral vegetal region extends to the posterior. The majority of evidence point to FGF signaling as being a posterior inducer. Dissociated cells from an ectodermal explant of Xenopus embryos were shown to adopt an anterior neural fate. However, additional FGF converted their fate to a posterior neural one [167,168,169], thus pointing to FGF signaling being posterior-inducing. Another set of experiments in Xenopus were performed under reduced levels of FGF activity; these were via FGF signaling block by N17Ras, a dominant-negative Ras mutant, and a truncated FGFR1 (XFD) [170]. Depleted FGF signaling by either N17Ras or XFD led to posterior markers not being expressed [170]. These results support the idea that FGF signaling is actively involved in posterior formation rather than in the anterior. Additional gain of function studies also in Xenopus drew similar conclusions; these were with overexpression of eFGF reported to induce a posteriorized phenotype with enlarging proctodeum and elevated expression level of posterior genes *xcad3*, *hoxB9*, and *hoxA7* [125,171]. In these instances of upregulation of posterior genes, the anterior specific factors were suppressed and resulted in small or truncated head formation [125,171].

Within the FGF family, there are also variants of FGFs that may have varying functions in axis development, although they are categorized under the same FGF signaling pathway. For example, FGF8 can generate the highest number of splice variants (FGF8a-h) among FGF family in mice, and these variants are subject to relative expression differences during development (reviewed [172]). FGF8a and FGF8b have been reported to have distinct instructive activity during brain development. In mice, FGF8a strongly induces the midbrain proliferation, wherein FGFb induces hindbrain fate specification, and the misexpression of these variants can revert developmental fate in various vertebrate models [172,173,174,175].

Both genes family of caudal homologs and Hox have been reported as the targets of FGF and Wnt signaling during AP elongation in vertebrates [125,171,176,177,178]. However, a growing body of evidence indicate that FGF signaling is not a solo regulator of AP patterning. Indeed several signaling pathways (such as FGF, RA, and Wnt) actively participate, and coordinate action for overall embryonic axis formation in vertebrate embryos [125,171,176,177,179,180,181,182]. One example is for MyoD, an important myogenic regulatory factor, which can be activated by *efgf* and *wnt8* during the Xenopus embryonic development [183,184]. Another example is in chick development with RA and FGF signaling cooperating for proper AP formation with RA targeting anterior most Hox genes whereby FGF targeting posterior most *hox* genes during neural tube development [185]. In mice and chicks, both RA and FGF signaling can also antagonistically interact during posterior development. The FGF signaling is active in the stem zone of undifferentiated cells in the posterior region for competence to become somitic mesoderm or spinal cord. Meanwhile, RA signaling is active in inducing somatic or neural differentiation in the transition zone [186,187]. Both RA and FGF pathways establish morphogen gradients in the anterior to posterior axis with RA concentrations being high at the anterior and FGF being high at the posterior end [186,187].

### 4.3. The LR Axis

The dorsoventral (DV) axis is followed by gastrulation organizing the anteroposterior (AP) axis. The left–right (LR) axis is formed and defined when in vertebrate embryos the anatomy or positions of visceral organs with respect to the midline become apparent. In mice, initiation of embryonic LR patterning may be defined in two crucial steps (reviewed [188]). In the first step, the node releases the asymmetric signals that diffuse toward the left side of lateral plate mesoderm (LPM) during neurula stage embryo. The second step is associated with gene activation in response to these asymmetric signals (reviewed [188]). The first asymmetric signal was reported as Sonic hedgehog (shh) protein in chick embryos [188]. To date, several signaling molecules have been characterized, particularly FGF8 shown to play an important role in LR axis formation across vertebrates [188,189,190,191]. In chicks, FGF8 induces the right-side determinant wherein shh has been reported with left-side inducer activity. In the right side, FGF8 inhibits the *nodal* and *pitx2* expression and activates *cSnR* (chick snail-related gene) expression [190]. Interestingly, in mice, FGF8 and shh exhibit the reverse effects as in the chick as FGF8 is left-side determining while shh promotes right-side determinants [190].

The most critical event in the symmetry-breaking event known to direct LR asymmetry in mammals is nodal flow. The nodal flow generally refers to leftward extracellular fluid movement powered by nodal cilia in the ventral nodes (review [192]). Nodal cilia biogenesis, releasing of VNPs (vesicular nodal parcels), KVs (Kupffer’s vesicle), and FGF signaling have all been reported to be actively involved in this process [2,193,194]. With regards to the FGF pathway, activation of key genes (*foxj1*, *rfx2*, *ier2*, *ift88*, and *fibp1*) responsible for functional nodal cilia are regulated by FGF signaling [195,196], and this plays several plausible roles throughout overall LR axis formation in vertebrates; however, the details of this regulation remain to be fully understood.

## 5. Regulation of FGF Signaling

Several agents have been discovered or experimentally developed that are relatively specific in modulating FGF signaling, and chemically, these include proteins and small molecules. Based on their activities, these modulators may be placed into three groups: The first includes the endogenous activator(s) as those agents having a positive or compensatory activity relative to FGF signaling. Second are the endogenous factors showing inhibitory or negative activity towards FGF signaling. Third includes the group of relatively small molecular-weight compounds, chemically synthesized to act as inhibitors and with use intended mostly for therapeutic or research applications. Prominently known endogenous activators/inhibitors of the FGF pathway were chosen to be briefly discussed.

### 5.1. Endogenous Protein Activators

A positive feedback regulatory mechanism plays an important role in embryonic and post embryonic development in vertebrates. In this process, several modulators may act as co-activators, interacting with a specific domain of an FGF or an FGFR; this interaction can in turn significantly accelerate or amplify FGF signaling. Notably, heparan sulfate proteoglycans (HSPGs) and glypicans are essential extracellular key modulators, which interact with a variety of growth factors including various FGFs and morphogens and their receptors [197]. These interactions can be positive as well as negative on target signaling with fine control being exerted in a spatiotemporal manner. Target signaling may include that for Wnt, Shh, and FGF pathways. Currently, several FGF positive modulators have been identified that include anosmin-1 (An1), Sef1, L1CAM, and FLRT3, and can alter FGF signaling at multiple levels (for more details, we refer the reader to the selected articles [197,198,199,200]) (Table 2). Certain small molecules have also been reported to induce FGF signaling. For example, 8-hydroxyquinoline sulfate and pyrithione zinc increases the expression of FGF target genes in zebrafish embryos [199]. However, as these reports provide a thoughtful explanation and clues to the regulatory mechanisms in FGF signaling, detailed mechanisms on the control of FGF signaling in development remain to be fully understood.

### 5.2. Endogenous Protein Inhibitors

A negative feedback mechanism is an essential regulatory strategy for nearly all signaling pathways and this is particularly important during embryonic development. FGF signaling has demonstrated as having an active functional role at multiple steps across embryonic development. To date, a group of endogenous proteins have been identified that show antagonist activity to FGF signals and are generally associated with the negative feedback loop in downregulating FGF signal transduction. One is Sprouty, an intracellular protein that interacts with Drk and Gap1, being components of Ras/MAPK pathway, and blocks their interaction [197,198]. Another is Sef, with a similar expression to various *fgf* genes, which is capable of interacting with the cytosolic domain of FGFR and blocking the intracellular effectors interacting with FGFR. An ectopic gain of function of *sef*, for example, has been shown to reduce phosphorylation of Raf1 and MEK1/2 [199]. Other agents that have been reported are Spred [200], Pyst1/Mkp3 [197], Dusp1 [92], and FRS2α/β [201], all endogenous FGF inhibitors/modulators. All of the above mentioned blockers of FGF signaling may have separate or overlapping targets (see Table 2) and are generally cytosolic effector proteins.

### 5.3. FGF Modulators in Developmental Biology

FGF modulators are essential in early embryogenesis as several studies have reported knockdown of various HSPG genes causing defective development in vertebrates and invertebrates (reviewed [202]). The initial reports from a vertebrate model, namely mouse, has the mutation of “lazy mesoderm” (*lzme*) gene where *lzme* encodes an enzyme required for glycosaminoglycan biosynthesis) causing defective mesoderm and endoderm migration [203]. Gene expression profiles demonstrated that FGF target genes expression are severely compromised in lzme mutant embryos, whereas the nodal and Wnt3 pathways remained normal [203]. Similar findings have been reported in Xenopus embryos, wherein morpholino-based knockdown of *gpc4* (Glypican 4) shows pleiotropic developmental aberrant phenotypes including defected primary axis and forebrain patterning [204]. A detailed discussion of FGF modulators, however, is beyond the focus of current review, and we refer the readers to the related articles [202,203,204,205,206,207].

## 6. Conclusions and Prospects

Aberrant FGF/FGFR signaling triggers numerous congenital disorders and many cancer types. Evidence obtained from gain-of-function/loss-of-function experiments demonstrates that proper FGF/FGFR function is required for correct embryonic development in vertebrates. The findings we discussed here suggest that various FGF ligands can trigger distinct germ layer specifications exclusively or in coordination with other signaling pathways. Genetic manipulation using recombinant techniques allowed us to investigate the genetic and functional diversity of FGF/FGFR signaling in early embryogenesis and postnatal development of vertebrates. The current understanding of the gene regulatory network (GRN) in embryonic development has made great progress, and we summarized some of the data within our line of investigation particularly for FGF signaling and its crosstalk with other signaling pathways. A number of particular FGFs were characterized that participate in early embryogenesis, and additional investigations for a more complete picture of their role in active GRN are required. The impressive array of additional modern techniques that have now become mainstream will be utilized for this purpose; these include single-cell RNA/DNA sequencing, efficient knock-in/knock-out technology of CRISPR, and characterizing hESCs along with vertebrate embryos. Taken together, this review provides an overview of the findings of the current research on embryonic development and lineage specification concerning FGF signaling in vertebrates.

## Figures and Tables

**Table 2:** Prominent protein modulators of FGF signaling with context and species dependent effects.

Modulators	Target	Effect on FGF Signaling
Anosmin-1	Extracellular domain of FGFR	Positive
L1CAM	Extracellular domain of FGFR	Positive
HSPGs	FGFs or extracellular domain of FGFR	Positive
FLRT3	Intracellular domain of FGFR	Positive
Sprouty	Grb/Raf	Negative
Sef’s/Spred/Pyst1/Dusp’s	Modification of several kinases activity like (MAPK, MEK, ERK, and PKB/Akt)	Negative

## Data Availability

Not applicable.
